# TCL1A-expressing B cells are critical for tertiary lymphoid structure formation and the prognosis of oral squamous cell carcinoma

**DOI:** 10.1186/s12967-024-05292-7

**Published:** 2024-05-19

**Authors:** Wenqiang Xie, Jinjin Lu, Yichen Chen, Xi Wang, Huanzi Lu, Qunxing Li, Nianqiang Jin, Jiankang He, Lingling Ou, Jia Ni, Yuqin Shen, Longquan Shao

**Affiliations:** 1 Stomatological Hospital, Southern Medical University, Guangzhou, 510280 PR China; 2 Department of Periodontics, Guangzhou Key Laboratory of Basic and Applied Research of Oral Regenerative Medicine, Affiliated Stomatology Hospital of Guangzhou Medical University, Guangzhou, 510182 Guangdong PR China; 3 Guanghua School of Stomatology, Guangdong Provincial Key Laboratory of Stomatology, Stomatological Hospital, Sun Yat-Sen University, Guangzhou, 510055 PR China; 4 Department of Stomatology, Sun Yat-sen Memorial Hospital, Sun Yat-sen University, Guangzhou, 510120 PR China

**Keywords:** B cells, Tertiary lymphoid structures, Oral squamous cell carcinoma, TCL1A

## Abstract

**Background:**

Oral squamous cell carcinoma (OSCC) is a malignant tumor with a poor prognosis. Traditional treatments have limited effectiveness. Regulation of the immune response represents a promising new approach for OSCC treatment. B cells are among the most abundant immune cells in OSCC. However, the role of B cells in OSCC treatment has not been fully elucidated.

**Methods:**

Single-cell RNA sequencing analysis of 13 tissues and 8 adjacent normal tissues from OSCC patients was performed to explore differences in B-cell gene expression between OSCC tissues and normal tissues. We further investigated the relationship between differentially expressed genes and the immune response to OSCC. We utilized tissue microarray data for 146 OSCC clinical samples and RNA sequencing data of 359 OSCC samples from The Cancer Genome Atlas (TCGA) to investigate the role of T-cell leukemia 1 A (TCL1A) in OSCC prognosis. Multiplex immunohistochemistry (mIHC) was employed to investigate the spatial distribution of TCL1A in OSCC tissues. We then investigated the effect of TCL1A on B-cell proliferation and trogocytosis. Finally, lentiviral transduction was performed to induce TCL1A overexpression in B lymphoblastoid cell lines (BLCLs) to verify the function of TCL1A.

**Results:**

Our findings revealed that TCL1A was predominantly expressed in B cells and was associated with a better prognosis in OSCC patients. Additionally, we found that TCL1A-expressing B cells are located at the periphery of lymphatic follicles and are associated with tertiary lymphoid structures (TLS) formation in OSCC. Mechanistically, upregulation of TCL1A promoted the trogocytosis of B cells on dendritic cells by mediating the upregulation of CR2, thereby improving antigen-presenting ability. Moreover, the upregulation of TCL1A expression promoted the proliferation of B cells.

**Conclusion:**

This study revealed the role of B-cell TCL1A expression in TLS formation and its effect on OSCC prognosis. These findings highlight TCL1A as a novel target for OSCC immunotherapy.

**Supplementary Information:**

The online version contains supplementary material available at 10.1186/s12967-024-05292-7.

## Introduction

With the process of social modernization, the incidence of cancer induced by chemical drugs, biological factors and physical factors has increased [1–3]. Cancer causes millions of deaths every year and has become a major burden for humans [4,5]. Oral squamous cell carcinoma (OSCC) is a malignant tumor originating from malignant mutations in epithelial cells and accounts for 90% of oral malignancies [6,7]. OSCC is also the sixth most common epithelial malignancy worldwide and has high morbidity and mortality rates [8]. Current treatments for OSCC include surgery, radiotherapy, and chemotherapy [7,9,10]. Although these traditional treatments have shown good curative effects, the five-year survival rate for OSCC patients is only 40-50% [11,12]. In addition, high doses of traditional treatments such as chemotherapy are often needed for OSCC, and this causes damage to the body and often leads drug resistance [13,14]. With the development of new chemotherapy drugs and drug delivery systems represented by nanoparticles, great progress has been made in the treatment of tumors [15–20]. However, the effectiveness of current chemotherapies for treating OSCC is still limited due to of the lack of therapeutic targets for OSCC. Therefore, new and improved treatment strategies are urgently needed.

Immunotherapy represents a promising new approach for OSCC treatment and has been demonstrated to have considerable efficacy [21–23]. However, the immunotherapy response rate is still low, which presents a challenge [24,25]. Numerous studies have indicated that tertiary lymphoid structures (TLS) play an important role in the response to immunotherapy [26,27]. However, the mechanism underlying the formation of TLS remains unclear [28]. Therefore, investigating the mechanism of TLS formation could enhance increase therapeutic efficacy in OSCC and prolong patient survival. TLS mainly consist of B cells, T cells, dendritic cells, natural killer cells, and endothelial cells [29,30]. Among these cells, B cells are the most abundant in TLS [27,31]. Although recent studies have highlighted the relationship between B cells and TLS formation [32,33], there is currently a scarcity of strategies for targeting B cells to intervene in TLS formation and tumor progression [34]. Therefore, it is important to explore the role of B cells in TLS formation and search for targets of immunotherapy-based interventions for OSCC.

By combining single-cell RNA sequencing and OSCC tissue analysis, we revealed that T-cell leukemia 1 A (TCL1A) is predominantly expressed in B cells and is downregulated in OSCC tissues vs. normal tissues. TCL1A is a known coactivator of AKT that has been extensively studied in T-cell leukemia and B-cell lymphoma [35–37]. However, the role of TCL1A in OSCC and TLS formation has not been elucidated. In the present study, we found that TCL1A was associated with OSCC prognosis and TLS formation. We then discovered that TCL1A-expressing B cells were located at the periphery of lymphatic follicles. Mechanistically, we found that TCL1A enhanced the antigen-presenting ability of B cells and promoted their proliferation, which may contribute to the formation of TLS. Our findings reveal a novel function of TCL1A in B cells, which may lead to the identification of a potential therapeutic target for OSCC.

## Materials and methods

### Patients and sample processing

To evaluate the relationship between TCL1A expression and TLS, we collected 29 OSCC tissues harvested from patients during surgery at the Hospital of Stomatology, Sun Yat-sen University. Tissue microarrays (TMAs) containing 146 OSCC tissue samples were obtained from the same hospital between January 2008 and December 2009 to assess the relationship between TCL1A expression and TLS formation. The follow-up period ended in April 2014. Peripheral blood samples were obtained from healthy donors. All the donors provided written informed consent. The research procedure involving the use of clinical specimens was approved by the Institutional Review Board of the Hospital of Stomatology, Sun Yat-sen University (number: KQEC-2020-09).

### Cell lines

The B lymphoblastoid cell line (GM12878) was obtained from Procell Life Science & Technology Co., Ltd. (Wuhan, China). These B lymphoblastoid cell line (BLCLs) were incubated in RPMI medium (Gibco cat# C11875500BT) supplemented with 10% fetal bovine serum (AUSGENEX cat# FBS500-S) and cultured at 37 °C with 5% CO_2_.

### Transfection

Lentiviral vectors for TCL1A overexpression were constructed by Tsingke Biotechnology Co., Ltd. (Beijing, China). After centrifugation, the culture medium was replaced with FBS-free Opti-MEM culture medium (Thermo Fisher cat# 3,198,070). The lentivirus (MOI = 100) and 8 µg/ml polybrene (Tocris cat# 7711/10) were added to Opti-MEM, and the cells were then centrifuged at 1000 × g for 1 h. They were subsequently inoculated into cell culture bottles for 1 day, after which the medium was replaced with RPMI medium supplemented with 10% FBS. The expression of green fluorescent protein (GFP) was observed after the transfected cells were cultured for 3 days. GFP positive cells were sorted via flow cytometry (BD Biosciences FACS Aria Fusion) and screened with 2 µg/ml puromycin (InvivoGen cat# ant-pr-1) after expansion.

### Quantitative real-time polymerase chain reaction

Total RNA from BLCLs was extracted using TRIzol reagent (Invitrogen cat# 15,596,018). A reverse transcription kit (TAKARA cat# RR036A) was used to synthesize cDNA. The expression of each gene was analyzed using a LightCycler 480 (Roche). The primer sequences for the internal standard control (β-actin) and TCL1A are listed in Table S1. All the samples were analyzed three times. The expression levels of all genes were calculated using the 2^−△△CT^ method.

### Immunohistochemistry (IHC)

After antigen retrieval, the TMAs were stained with a TCL1A antibody (Santa Cruz Biotechnology cat# sc-393,436) at 4 °C. Subsequently, the TMAs were stained with a horseradish peroxidase-conjugated secondary antibody (Gene Tech cat# GK500710). Hematoxylin was used for nuclear labeling. The TMA data were obtained using a slide scanner (Leica Aperio AT2). Four random regions were selected from each sample under a low-power lens. TCL1A expression in the images was quantified by measuring the integrated density using ImageJ software. The results of the TMAs were reviewed by two certified pathologists.

### Pathological staining and analysis

Paraffin-embedded OSCC tissue slices were stained with hematoxylin and eosin (H&E). Images of the H&E stained sections were acquired using a slide scanner (Leica Aperio AT2). Subsequently, two certified pathologists classified these images as TLS-positive or TLS-negative.

### Immunofluorescence

After antigen retrieval, paraffin-embedded tissue slices from OSCC patients were blocked with goat serum after permeabilization with 0.2% Triton X-100. All slices were then incubated with an anti-CD19 antibody (CST cat#3574), anti-TCL1A antibody (Santa Cruz Biotechnology cat# sc-393,436), anti-CR2 antibody (Affinity cat# DF7770), or anti-KI67 antibody (CST cat#9129) overnight at 4 °C. All slides were imaged under a fluorescence microscope (Olympus FV3000) after incubation with secondary antibodies (Earthox cat# E032210, cat# E032420).

### Multiplex immunohistochemistry (mIHC)

An Opal Fluorescent IHC Kit (PerkinElmer cat# NEL811001KT) was used to perform mIHC staining. OSCC tissue slices were incubated overnight with primary antibodies at 4 °C. The primary antibodies used were as follows: anti-CD4 (CST cat#25,229), anti-CD19 (CST cat#3574), anti-CD8 (CST cat# 98,941), anti-TCL1A (Santa Cruz Biotechnology cat# sc-393,436), and anti-PNAD (Abcam cat# ab111710). The OSCC slices were incubated with secondary antibody for 10 min at room temperature. After that, all slices were incubated with dye (Opal TSA) for 20 min at room temperature. The Opal detection fluorophores used were as follows: CD8-Opal 690, CD4-Opal 650, PNAD-Opal 620, CD19-Opal 570, and TCL1A-Opal 520. All mIHC images were captured using TissueFAXS Imaging software (TissueGnostics v7.134).

### Isolation of human blood B cells and dendritic cells

Peripheral blood from healthy donors was used to isolate peripheral blood mononuclear cells (PBMCs) with Ficoll-Paque Plus (GE Healthcare cat# 17,144,003). B cells were sorted from PBMCs using the MojoSort™ Human CD19 Selection Kit (BioLegend cat#480,105). Briefly, 10^8^ cells were blocked to prevent Fc receptor binding and incubated with a cocktail of biotin-conjugated antibodies and anti-biotin microbeads. The labeled cells were sorted using magnetic columns. Dendritic cells were sorted from PBMCs using an enrichment kit (Miltenyi Biotec cat#130-100-777). The sorting method for dendritic cells was similar to that for B cells.

### Drug treatment

To upregulate TCL1A expression, the BLCLs and purified B cells were treated with estradiol [38]. Prior to treating the cells with estradiol, the BLCLs or B cells were cultured in media supplemented with 5% FBS for 1 day. The cells were cultured in serum-free medium for an additional 1 day. Subsequently, all the cells were treated with 0.1 nM estradiol (Sigma cat# E2758) for 1 day.

### Flow cytometry

Fc receptor blocking was performed for B cells or BLCLs. The cells were stained with antibodies in the dark. Before TCL1A (eBioscience cat# 12-6699-42) or KI67 (Biolegend cat# 350,513) staining, Ghost Dye Red 780 staining was performed, followed by staining for surface markers for 30 min at 4 °C in the dark. The cell membranes and nuclear membranes were permeabilized using a kit (Tonbo Biosciences cat# TNB-0607-KIT) prior to TCL1A or KI67 staining. A DxFLEX instrument (Backman) was used to perform flow cytometry, and the data were analyzed using FlowJo 10.5.3 software. In addition, the fluorescence minus one method was used to establish gates.

### Trogocytosis assay

Before the trogocytosis assay, the sorted dendritic cells were stained with CD11c (Biolegend cat# 980,604) and HLA-DR (Biolegend cat# 980,420). The sorted B cells were divided into two groups: one group was treated with estradiol, and the other group was treated with the same amount of vehicle. Two groups of B cells (2 × 10^5^) were mixed with dendritic cells (3 × 10^5^). The mixed cells were cocultured in 96-well U-bottom cell culture plates in RPMI medium supplemented with 10% FBS for 2 h at 37 °C after centrifugation at 150 g for 1 min to promote contact between the two cell types. After coculture, CD19 (Biolegend cat# 982,402) staining was performed. Flow cytometry was used to detect the CD11c and HLA-DR fluorescence of B cells.

### Bioinformatic analysis

RNA-seq data for the OSCC patient cohort were obtained from The Cancer Genome Atlas (TCGA) [39]. In brief, the survival data (version: 07-19-2019) and RNA-seq data (version: 07-19-2019) of head and neck cancer (HNSC) patients were downloaded from the University of California–Santa Cruz Xena. A total of 359 OSCC samples were identified by screening of the HNSC samples. All RNA-seq data from OSCC tissues were divided evenly into two groups (TCL1A low and TCL1A high) according to TCL1A expression, and overall 5-year survival was analyzed via Kaplan‒Meier analysis. A heatmap was generated with the R package Pheatmap. The TLS signatures were the gene sets reported in previous articles for identifying TLS [40].

Single-cell RNA sequencing data of 13 tissues and 8 adjacent normal tissues from OSCC patients were obtained from “https://bigd.big.ac.cn/gsa-human/browse/HRA001006” and analyzed using the R package Seurat. The FindAllMarkers() function was used to search for the differential marker genes for each cell cluster. We then determined the cell types according to multiple differential marker genes. The module score of each gene signature was used to label the cells by color on the UMAP projection generated by Seurat. To analyze the expression of TCL1A and other marker genes in B-cell types, B cells and plasma cells were extracted from the integrated dataset. The B-cell type subset was subjected to principal component analysis (PCA), and different cell types were clustered according to the top 30 PCs. The FindClusters() function of Seurat was used to identify the types of B cells. Dot plots, violin plots, and feature plots were drawn using the Dotplot, Vlnplot, and Featureplot functions, respectively.

### Cell cycle detection

BLCLs were collected and resuspended in 1 ml of PBS. Then, 3 ml of precooled anhydrous ethanol was added to fix the cells for 24 h. Cell cycle progression was assessed using a cell cycle detection kit (MCE cat# HY-K1071), and the results were detected via flow cytometry (Backman DxFLEX). The data were analyzed using FlowJo 10.5.3 software. The proportions of cells in the G1 phase, G2 phase and S phase were determined.

### Statistical analysis

Kaplan‒Meier curves were constructed to analyze the 5-year overall survival of the OSCC patients in the TMA and TCGA cohorts. Cox regression analysis was used to perform a multivariate analysis of the TMA data. The receiver operating characteristic (ROC) curve of TCL1A expression was used to validate its potential value in identifying TLS in OSCC patients. Unpaired Student’s *t* tests (two-group comparisons) were used for data analysis.

## Results

### TCL1A is predominantly expressed in B cells and associated with a better prognosis for OSCC patients

**Fig. 1:**
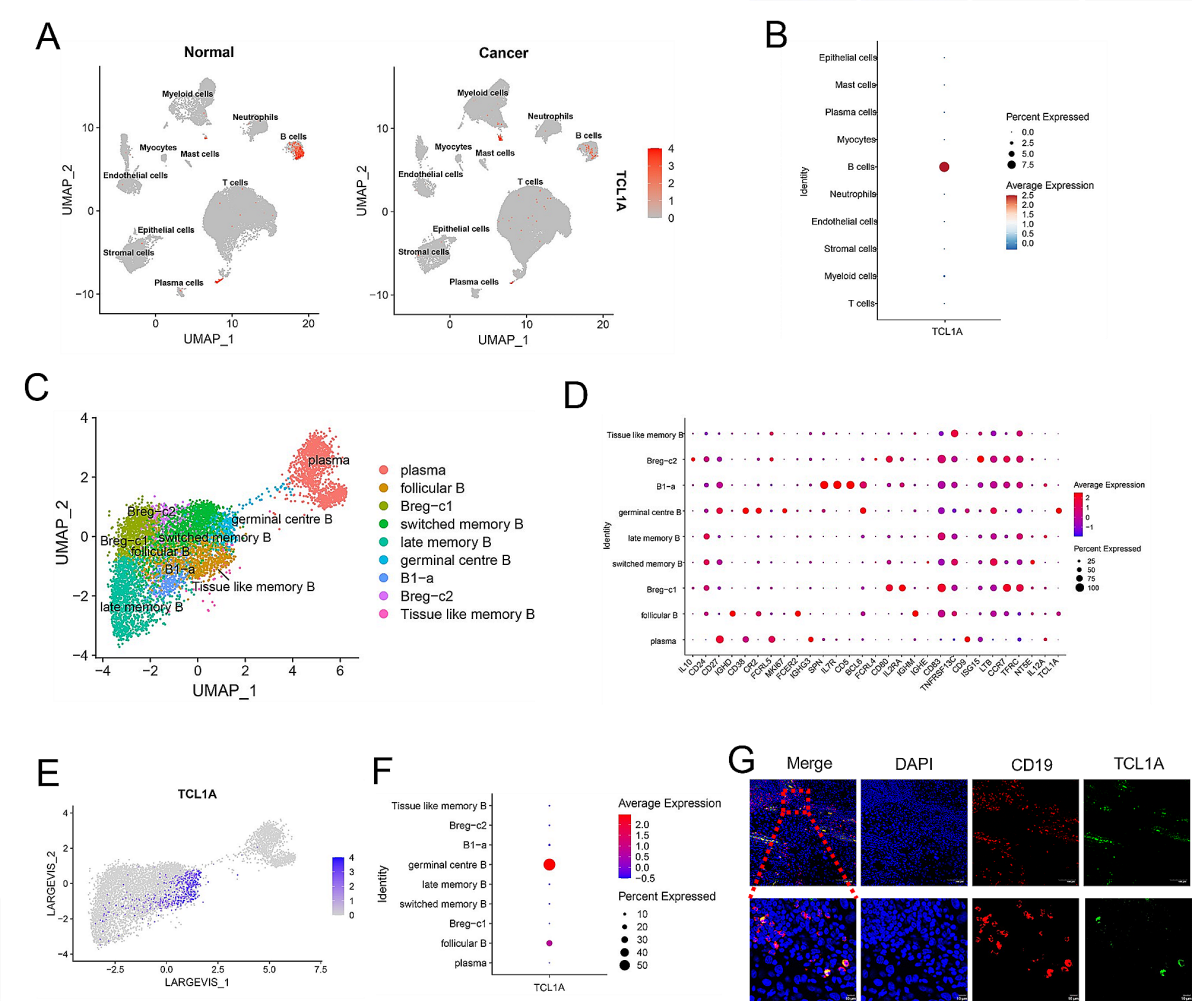
TCL1A is downregulated in OSCC tissues compared with normal tissues and is predominantly expressed in B cells. **A** Feature plot of single-cell RNA sequencing data showing that TCL1A is predominantly expressed in B cells and downregulated in OSCC tissues compared to normal tissues. **B** Dot plot showing that TCL1A is predominantly expressed in B cells. **C** UMAP plot showing that B cells were extracted and regrouped into different subsets. **D** Dot plot showing the differentially expressed genes among B-cell subsets. **E, F** Feature plot and dot plot showing that TCL1A is expressed mainly in germinal center B cells, followed by follicular B cells. **G** IF staining showing the expression levels of CD19 (red) and TCL1A (green) in OSCC tissues. Scale bars: 100 μm (upper); 10 μm (lower)

To explore the role of B cells in the progression of OSCC, we analyzed the differentially expressed genes in B cells between OSCC tissues and adjacent normal tissues by single-cell RNA sequencing analysis. The results showed that TCL1A was predominantly expressed in B cells and was significantly downregulated in OSCC tissues compared to normal tissues (Fig. 1A, B). We then extracted the B cells and reclustered them into 9 subsets (Fig. 1C, D). Further analysis revealed that TCL1A was expressed mainly in germinal center B cells, followed by follicular B cells (Fig. 1E, F). Immunofluorescence analysis of OSCC tissues further verified that TCL1A was expressed mainly in B cells (Fig. 1G).

**Fig. 2:**
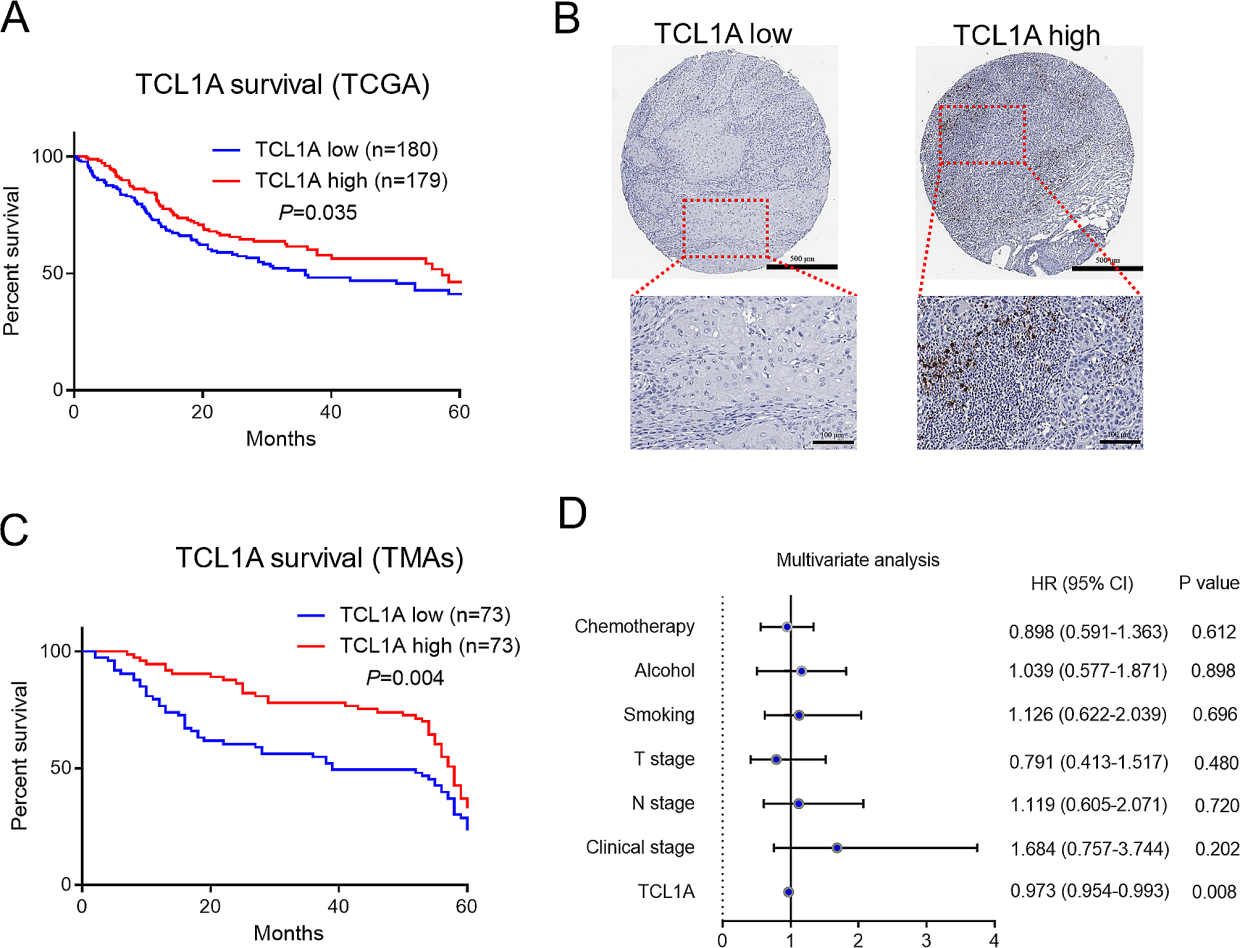
TCL1A expression is related to favorable prognosis in OSCC patients. **A** Kaplan-Meier survival curves showing that TCL1A upregulation is significantly associated with improved OS in patients with OSCC (TCGA database). **B** Representative IHC images of tissue microarrays (TMAs). Scale bars: 500 μm (upper); 100 μm (lower). **C** Kaplan-Meier survival curve showing that TCL1A upregulation is significantly associated with improved overall survival in 146 OSCC patients according to IHC staining. **D** Cox regression analysis showing the multivariate analysis of factors related to overall survival in 146 OSCC patients in the TMA cohort

We subsequently investigated the correlation between TCL1A expression in OSCC samples and prognosis. Using the TCGA dataset, OSCC samples were divided into two groups based on their TCL1A expression levels (TCL1A-low and TCL1A-high). Analysis of the 5-year overall survival rates of these two groups revealed that OSCC patients with high TCL1A expression exhibited a significantly improved prognosis (Fig. 2A). To further validate these findings, we performed immunohistochemical staining for TCL1A on tissue microarrays (TMAs) obtained from 146 OSCC patients. Similar to the TCGA results, the patients providing TMA samples were also evenly divided into TCL1A-high and TCL1A-low groups (Fig. 2B). Kaplan‒Meier survival analysis demonstrated that OSCC patients in the TCL1A-high subgroup had a greater 5-year overall survival rate than did those in the TCL1A-low subgroup (Fig. 2C). Additionally, Cox regression analysis including variables such as chemotherapy, alcohol consumption, smoking status, T stage, N stage, clinical stage, and TCL1A expression revealed that low TCL1A expression was a risk factor (Fig. 2D). These results collectively demonstrate the association of TCL1A with improved prognosis in patients with OSCC.

### The expression of TCL1A is associated with TLS formation in OSCC tissues

**Fig. 3:**
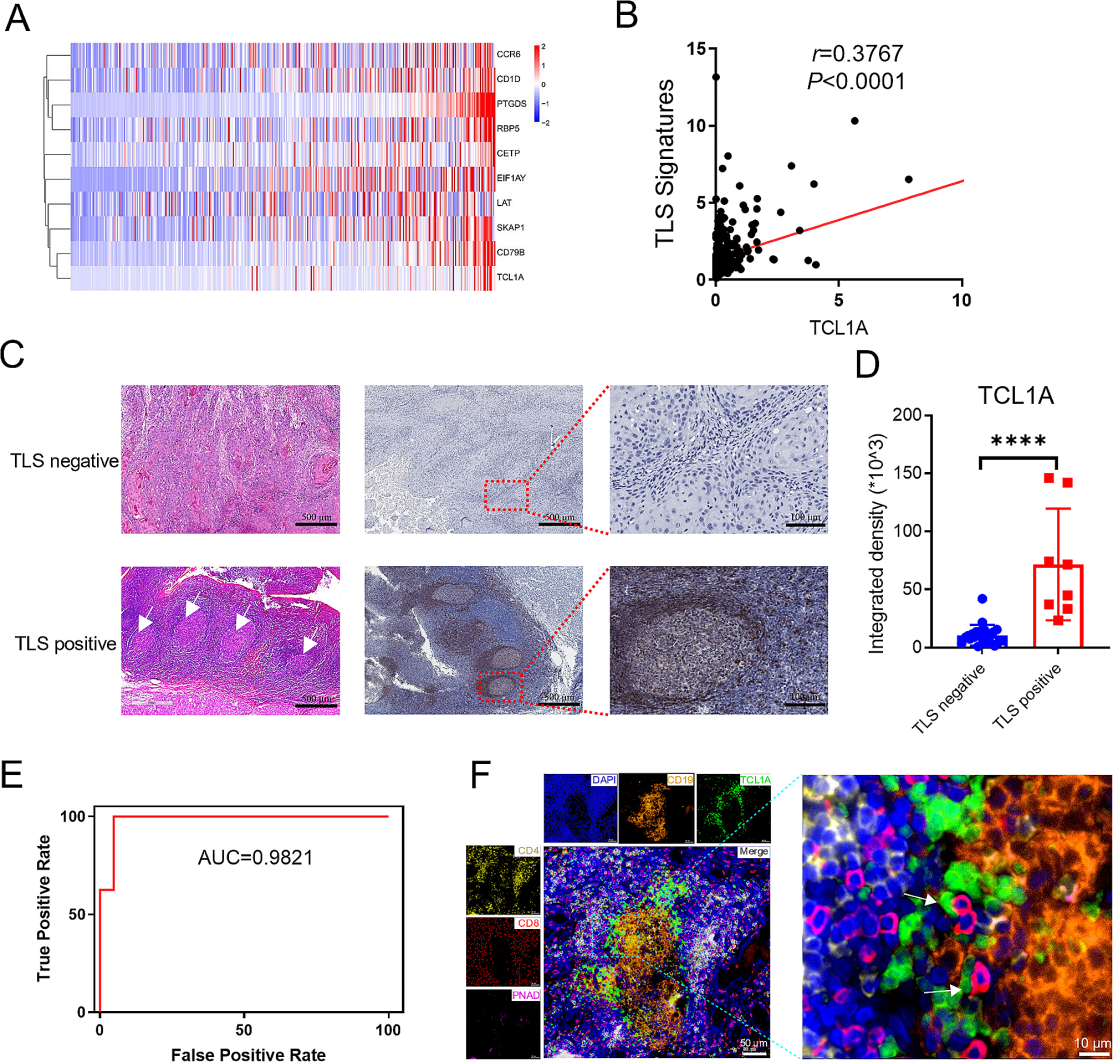
TCL1A is highly expressed in tertiary lymphoid structure (TLS)-positive tissues from OSCC patients. **A, B** The expression of TCL1A is positively correlated with the enrichment of TLS signatures (*EIF1AY*, *CETP*, *CCR6*, *CD79B*, *LAT*, *PTGDS*, *CD1D*, *RBP5*, and *SKAP1*) in OSCC samples from the TCGA. **C** Representative images of H&E and immunohistochemistry (IHC) staining of OSCC tissues (white arrows indicate TLS). Scale bar: 500 μm (left); 100 μm (right). **D** Quantitative analysis showing the upregulation of TCL1A in TLS-positive tissues from OSCC patients according to the intensity of IHC staining. **E** The ROC curves for the TCL1A expression analysis validated its potential value in identifying TLS in OSCC patients. ****P < 0.0001. **F** mIHC staining showing the spatial localization of different cells in the TLSs of OSCC tissues

Since germinal center B cells and follicular B cells are closely associated with the formation of tertiary lymphoid structures (TLS), the expression of TCL1A might be correlated with TLS formation. To test this hypothesis, we analyzed the gene expression patterns of oral squamous cell carcinoma (OSCC) in the TCGA cohort using heatmaps and observed a positive correlation between the expression of TCL1A and TLS signatures (Fig. 3A). Correlation analysis further demonstrated a relationship between the expression of TCL1A and TLS signatures (Fig. 3B). Additionally, TLS positivity in OSCC tissues was confirmed by pathological staining. We divided the collected OSCC samples into TLS-positive and TLS-negative groups according to pathological conditions and then assessed the expression of TCL1A in the two groups. Immunohistochemical staining revealed that the expression of TCL1A in the TLS-positive group was significantly greater than that in the TLS-negative group (Fig. 3C, D). Furthermore, receiver operating characteristic (ROC) curve analysis revealed that the expression of TCL1A could serve as a robust biomarker for the presence of TLS in OSCC tissues, with area under the curve (AUC) values exceeding 0.9 (Fig. 3E). We further explored the spatial distribution of TCL1A-expressing B cells in TLS. mIHC staining of OSCC samples revealed that TCL1A-expressing B cells were predominantly located at the periphery of lymphatic follicles and at the interface between the T-cell and B-cell regions (Fig. 3F).

### TCL1A-expressing B cells undergo trogocytosis mediated by CR2 and exhibit increased proliferative ability

**Fig. 4:**
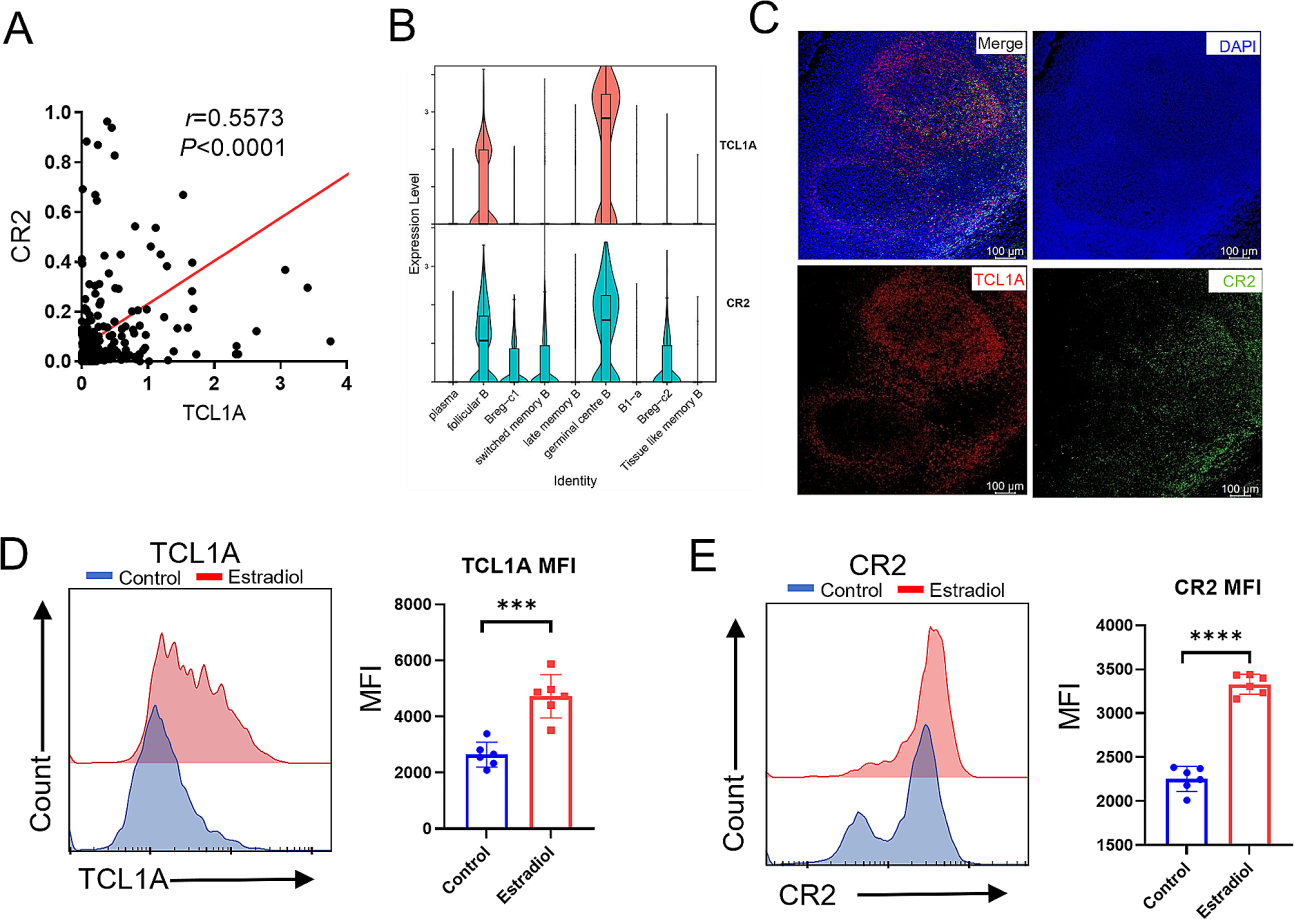
CR2 expression in B cells increases after TCL1A expression is upregulated. **A** The expression of TCL1A is positively correlated with that of CR2 in OSCC samples from the TCGA database. **B** Violin plot showing that CR2 is highly expressed in TCL1A-expressing B cells. **C** IF staining showing the expression of TCL1A (red) and CR2 (green) in TLSs from OSCC tissue (scale bars: 100 μm). **D, E** Flow cytometry analysis showing that the proportions of TCL1A and CR2 cells increased after B cells were treated with estradiol. **P < 0.01; ***P < 0.001; ****P < 0.0001

**Fig. 5:**
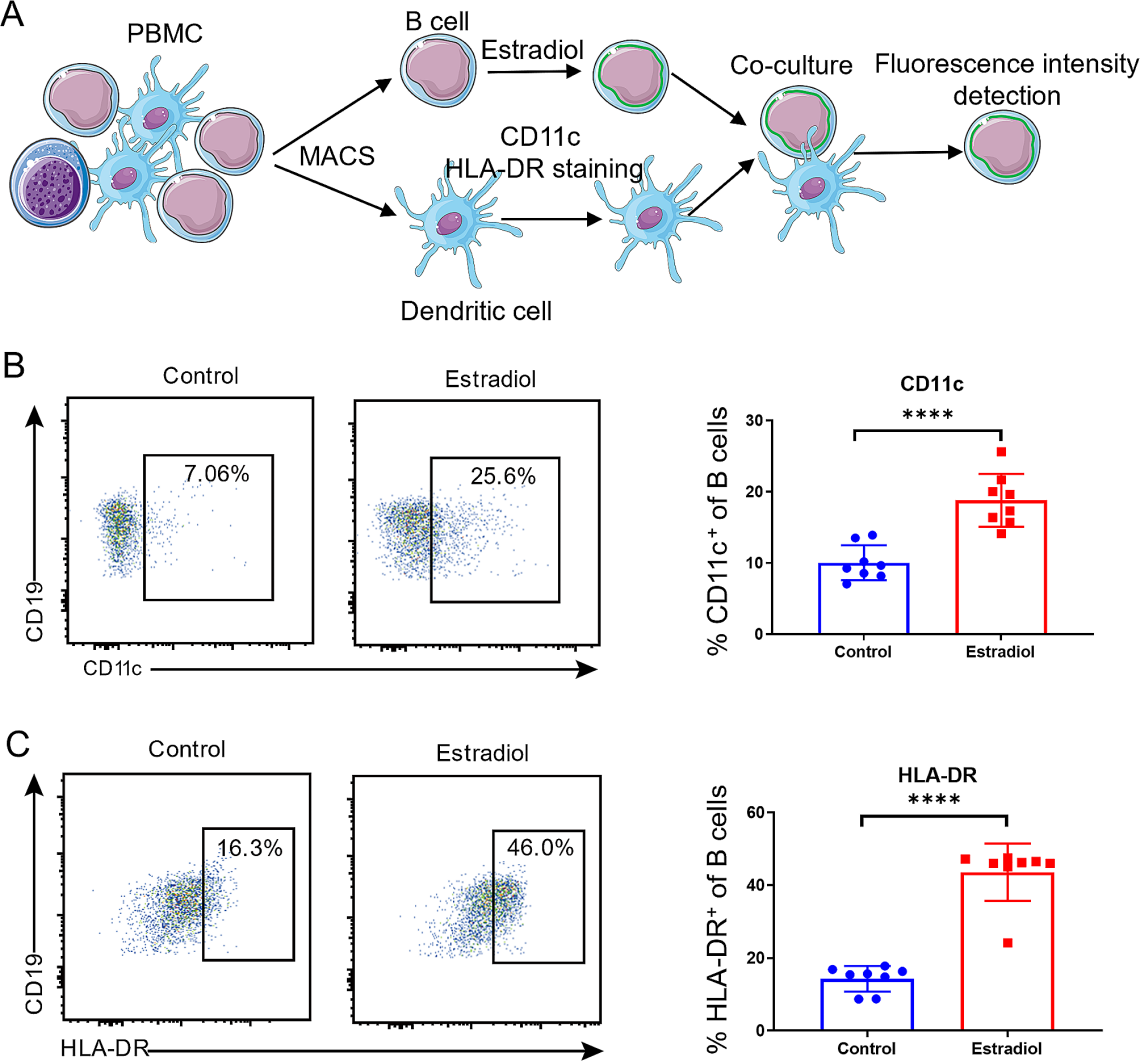
The trogocytosis effect of B cells is promoted after TCL1A expression is upregulated. **A** Flowchart illustrating the methodology employed to detect the trogocytosis effect of TCL1A^+^ B cells on dendritic cells. MACS: magnetic activated cell sorting. **B, C** Flow cytometry analysis demonstrated that B cells treated with estradiol promoted the trogocytosis-mediated transfer of CD11c and HLA-DR on dendritic cells

We then investigated the potential role of TCL1A-expressing B cells in TLS formation. By analyzing the OSCC sequencing data from the TCGA database, we found that TCL1A expression was associated with CR2 expression (Fig. 4A). This correlation was further supported by single-cell RNA sequencing data from OSCC patients (Fig. 4B). Immunofluorescence analysis of OSCC tissues also confirmed that TCL1A-positive cells were colabeled with CR2 (Fig. 4C). Previous studies have shown that estradiol can upregulate the expression of TCL1A in B cells [38]. B cells were treated with estradiol and analyzed using flow cytometry (Figure S1). The results revealed an increase in the expression of TCL1A and CR2 in B cells after estradiol treatment (Fig. 4D, E). A previous study reported that B cells can exert a trogocytosis effect on dendritic cells through CR2 [41]. To investigate whether TCL1A-expressing B cells can also exert this effect, we sorted B cells and dendritic cells from PBMCs. B cells were stimulated with estradiol, and fluorescent-labeled antibodies were used to label CD11c and HLA-DR on dendritic cells. The two groups of cells were then cocultured to detect the fluorescence intensity of the B cells (Fig. 5A). The results revealed that the fluorescence intensity of B cells treated with estradiol was greater than that of untreated B cells (Fig. 5B, C). These findings suggest that TCL1A-expressing B cells can exert a trogocytosis effect on dendritic cells through the upregulation of CR2, which may increase the antigen-presenting capacity of B cells.

Single-cell RNA sequencing data suggested that TCL1A was expressed mainly in germinal center B cells, which are a group of rapidly proliferating B cells. We further investigated the effect of TCL1A on B-cell proliferation. First, we found a positive correlation between the expression of TCL1A and that of KI67 in B cells from single-cell RNA sequencing data of OSCC (Figure S2A). Immunofluorescence results also confirmed that TCL1A-positive cells in OSCC tissues were colabeled with KI67 (Figure S2B). Flow cytometry analysis revealed that KI67 expression and B-cell proliferation were promoted after the expression of TCL1A was upregulated (Figure S2C, D). These findings indicate that the upregulation of TCL1A promotes the proliferation of B cells.

To investigate whether TCL1A-expressing B cells can also exert this effect, we sorted B cells and dendritic cells from PBMCs. B cells were stimulated with estradiol, and fluorescent-labeled antibodies were used to label CD11c and HLA-DR on dendritic cells. The two groups of cells were then cocultured to detect the fluorescence intensity of the B cells (Fig. 5A). The results revealed that the fluorescence intensity of B cells treated with estradiol was greater than that of untreated B cells (Fig. 5B, C). These findings suggest that TCL1A-expressing B cells can exert a trogocytosis effect on dendritic cells through the upregulation of CR2, which may increase the antigen-presenting capacity of B cells.

Single-cell RNA sequencing data suggested that TCL1A was expressed mainly in germinal center B cells, which are a group of rapidly proliferating B cells. We further investigated the effect of TCL1A on B-cell proliferation. First, we found a positive correlation between the expression of TCL1A and that of KI67 in B cells from single-cell RNA sequencing data of OSCC (Figure S2A). Immunofluorescence results also confirmed that TCL1A-positive cells in OSCC tissues were colabeled with KI67 (Figure S2B). Flow cytometry analysis revealed that KI67 expression and B-cell proliferation were promoted after the expression of TCL1A was upregulated (Figure S2C, D). These findings indicate that the upregulation of TCL1A promotes the proliferation of B cells.

To further validate these results, the BLCLs were treated with estradiol to upregulate the expression of TCL1A (Figure S3A). Moreover, flow cytometry analysis revealed that the expression of CR2 and KI67 increased (Figure S3B, C). Additionally, the percentage of fast-proliferating BLCLs (CellTrace^−^) increased after TCL1A was upregulated, indicating an increase in cell proliferation (Figure S3D).

### Overexpression of TCL1A in B cells promotes CR2 expression and cell proliferation

**Fig. 6:**
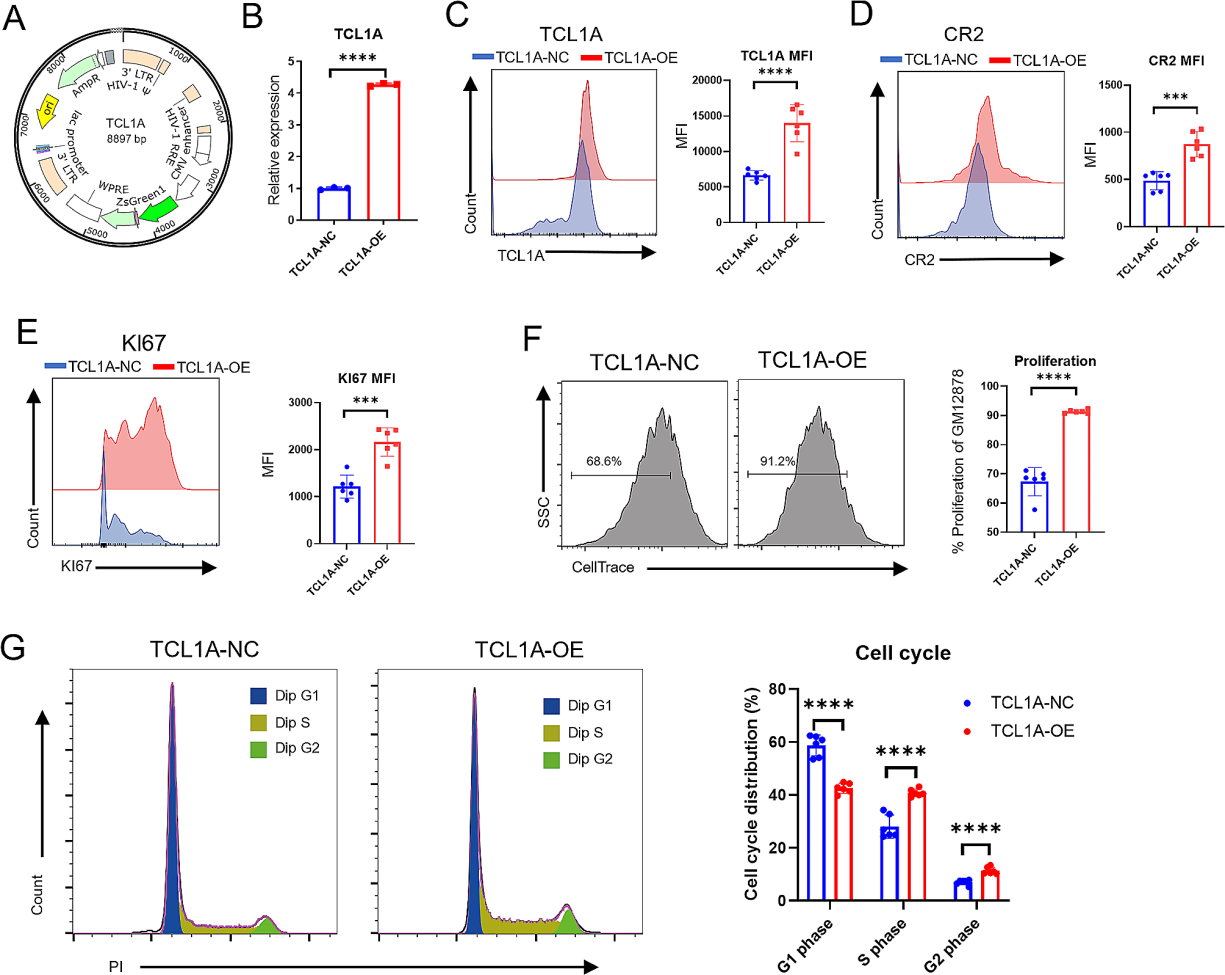
CR2 expression is increased after TCL1A overexpression in BLCLs. **A** Construction of lentivirus for TCL1A overexpression. **B** RT-qPCR results showing that the expression level of TCL1A in the overexpression group (TCL1A-OE) was greater than that in the negative control group (TCL1A-NC). **C-E** Flow cytometry analysis revealing an increase in the expression of TCL1A, CR2, and KI67 after BLCLs were transduced with lentivirus. **F** Flow cytometry analysis showing that the percentage of fast-proliferating (CellTrace^−^) cells among BLCLs increased after TCL1A overexpression. **G** Flow cytometry analysis showing the cell cycle distribution of TCL1A-NC and TCL1A-OE cells. Representative results are shown (left). The results of the quantitative analysis of the population of BLCLs that proliferated quickly are shown (right). Each data point represents an individual subject. *P < 0.05; ***P < 0.001; ****P < 0.0001

To investigate the direct regulatory effect of TCL1A on CR2 expression and proliferation in B cells, we generated a lentiviral vector overexpressing TCL1A and transduced it into BLCLs (Fig. 6A). Transfected B cells were sorted based on GFP expression and screened using puromycin. The fluorescence of the transfected cells was confirmed by flow cytometry and laser confocal microscopy (Figure S4A, B). The efficiency of TCL1A overexpression was validated using RT‒qPCR and flow cytometry (Fig. 6B, C). Further investigation revealed that CR2 and KI67 expression was enhanced in BLCLs after TCL1A was overexpressed (Fig. 6D, E). Additionally, the percentage of fast-proliferating BLCLs (CellTrace^−^) increased after TCL1A was overexpressed, which indicated an increase in the proliferation of the B-cell line (Fig. 6F). We then explored the effect of TCL1A overexpression on the cell cycle in BLCLs. The results showed that the proportion of cells in the G1 phase decreased, while the proportion of cells in the S phase and G2 phase increased after TCL1A overexpression.

**Fig. 7:**
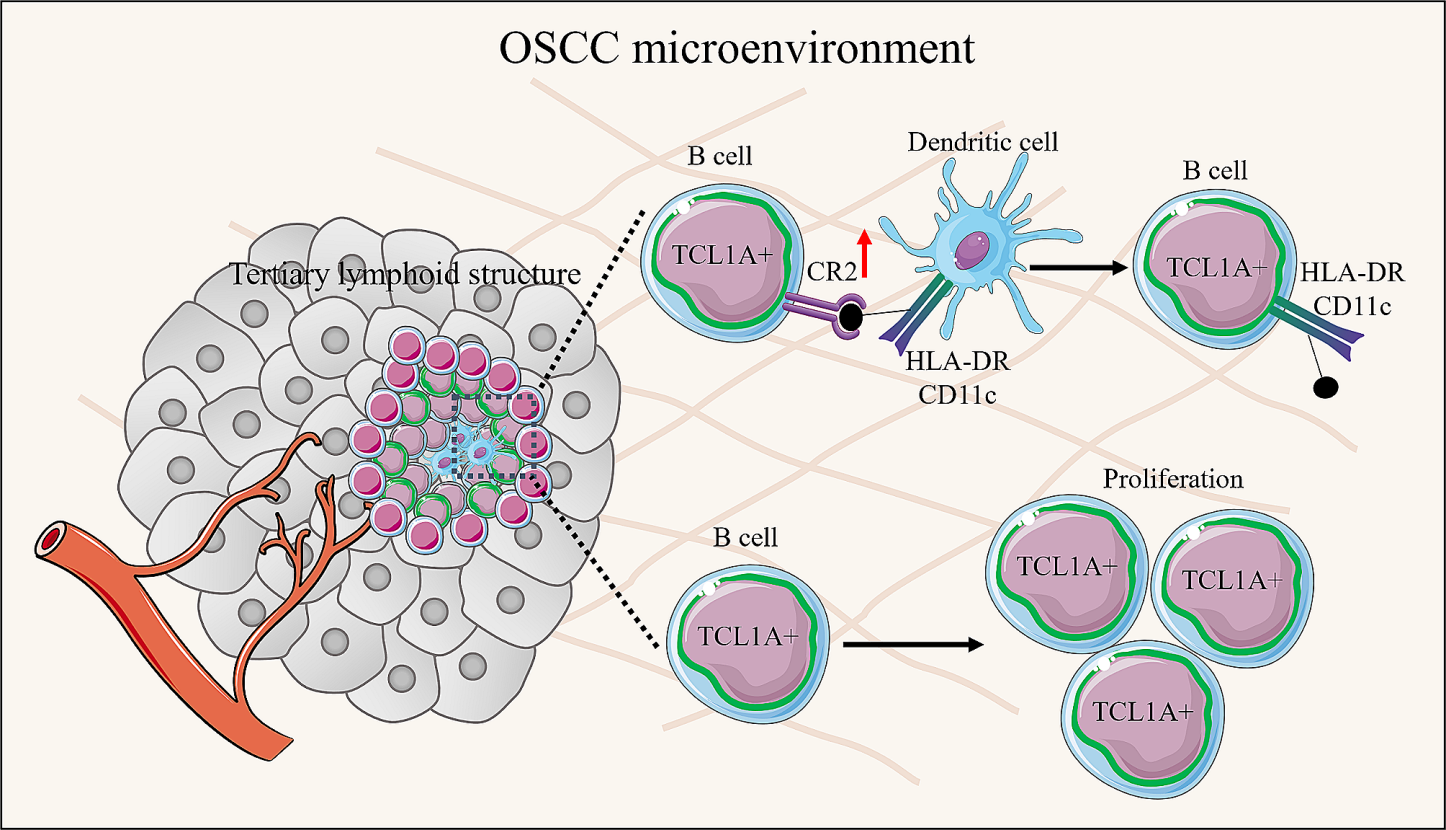
Working model: The role of TCL1A-expressing B cells in the tertiary lymphoid structures of OSCC

## Discussion

In the present study, we utilized single-cell RNA sequencing data, TCGA data and clinical samples to investigate the relationship between TCL1A expression and prognosis in OSCC patients and the role of TCL1A in TLS formation. Our findings revealed that TCL1A was associated with TLS formation in OSCC patients and a better prognosis in patients with OSCC. Mechanistically, TCL1A enhanced the trogocytosis effect of B cells by upregulating the expression of CR2 and promoting the proliferation of B cells (Fig. 7). These findings indicated that targeting TCL1A in B cells could be a novel approach for improving OSCC immunotherapy efficacy.

Although recent studies have shown that CCR6, CD1D, CD79B, CXCL13, and L1CAM are related to the formation of TLS, TLS still lack definite markers [40,42–44]. In the present study, we found that TCL1A could serve as a potential marker of TLS formation in OSCC tissues. Additionally, since TLS formation is closely associated with tumor immunotherapy efficacy, the expression level of TCL1A could serve as a basis for evaluating the efficacy of immunotherapy in OSCC patients. However, TCL1A levels in other tumors, such as T-cell leukemia and B-cell lymphoma, have been demonstrated to be associated with a poor tumor prognosis [36,45]. The discrepancies in our findings can be attributed to the differential cellular localization of TCL1A. In T-cell leukemia and B-cell lymphoma, TCL1A is primarily expressed in tumor cells, where it promotes tumor cell proliferation and leads to a poor prognosis [35,46,47]. Conversely, in OSCC, TCL1A is predominantly expressed in B cells rather than in tumor cells and may enhance the immune response against OSCC by augmenting the proliferation and antigen-presenting ability of B cells. The consistent effect of TCL1A on the proliferation of tumor cells in T-cell leukemia and B-cell lymphoma further supports its universal ability to enhance cell proliferation. Our findings provide a potential marker for predicting TLS formation and OSCC prognosis.

There have been numerous reports on the relationship between T cells and tumors, but the role of B cells in tumors has often been overlooked [48,49]. Currently, the association between B cells and tumor prognosis, indicating the heterogeneity of B cells within tumors, is controversial [49]. B cells infiltrating tumor tissues can be classified into three categories based on their functions: regulatory B cells, antibody-secreting B cells, and antigen-presenting B cells [50,51]. B cells primarily exert antitumor effects via through antibody secretion and antigen-presenting functions [32,52,53]. However, B cells can also promote tumor growth by inhibiting immune responses through regulatory B cells [50]. Before B cells can perform their antibody-secreting and antigen-presenting functions, they need to undergo proliferation and antigen activation. Our study further revealed that upregulation of TCL1A expression led to increased proliferation of B cells and enhanced expression of CR2. CR2 provides a secondary signal for B-cell activation, which plays an important role in the immune response of B cells [54]. These findings suggested that TCL1A-expressing B cells are a subset of rapidly proliferating and easily activated cells. Initial activation of B cells in TLS typically occurs in the periphery of lymphoid follicles, and our mIHC analysis of TLS confirmed that TCL1A-expressing B cells were predominantly located in the peripheral regions of lymphoid follicles. This spatial distribution further supports the notion that TCL1A-expressing B cells are primed for activation. Moreover, a recent study demonstrated that B cells can acquire MHC II by trogocytosing dendritic cells through CR2, thereby obtaining antigen-presenting capabilities [41]. Consistent with previous findings, TCL1A-expressing B cells can also trogocytose dendritic cells through CR2. These results suggested that TCL1A-expressing B cells in OSCC could undergo rapid proliferation and acquire antigen-presenting capacity via CR2. Several studies have shown that CR2-positive B cells are related to TLS formation and cancer prognosis [55,56], but the underlying mechanism has not been explored in depth. The findings of this study provide a reasonable explanation for this phenomenon and provide a novel target for cancer treatment. In addition, the current focus of tumor immunotherapy is the regulation of immune checkpoints targeting T cells. Such methods tend to cause excessive activation of T cells throughout the body, resulting in autoimmune diseases [57]. In this study, targeting of TCL1A in B cells was shown to enhance the antitumor effects of B cells, so this approach has the potential to replace existing therapies.

Additionally, our study demonstrated that estradiol can upregulate the expression of TCL1A in B cells, thereby enhancing CR2 expression and promoting B-cell proliferation. These results suggested that estradiol may play a potential role in augmenting the antitumor immune response of B cells in OSCC and could be combined with existing immunotherapy methods to improve treatment efficacy. However, previous studies have shown that estradiol also promotes tumor cell proliferation in OSCC, which worsens the prognosis of the disease [58,59]. Therefore, the development of a precise drug delivery system for the targeted delivery of estradiol to B cells is warranted for the treatment of OSCC. In recent years, many novel nanomaterials have been developed as drug delivery systems to treat tumors. These drug delivery systems show great safety and targeting ability [19]. Therefore, the combination of nanomaterials targeting tumor-infiltrating B cells with estradiol is expected to induce antitumor effects in OSCC by regulating the expression of TCL1A in B cells.

This study has several limitations that need to be addressed in future investigations. For instance, it is crucial to experimentally confirm whether the expression of TCL1A in B cells triggers TLS formation in OSCC tissues. This can be accomplished by conducting experiments on B cells from mice with conditional knock-in of TCL1A. Furthermore, the underlying mechanism by which TCL1A upregulates CR2 expression in B cells requires further investigation. Exploring this mechanism will provide valuable insights into the role of TCL1A in the formation of TLS.

In summary, this study revealed that TCL1A could serve as a marker for predicting TLS formation and OSCC prognosis. Furthermore, upregulation of TCL1A in B cells could enhance their proliferation and trogocytosis effect. Based on the various functions of TCL1A, targeting B-cell TCL1A is anticipated to emerge as a crucial therapeutic approach for managing OSCC.

### Electronic supplementary material

Below is the link to the electronic supplementary material.

Supplementary Material 1

## Data Availability

Single-cell RNA sequencing data of OSCC were available from “https://bigd.big.ac.cn/gsa-human/browse/HRA001006”. RNA-seq data of the OSCC patient cohort from The Cancer Genome Atlas (TCGA) were obtained from the University of California–Santa Cruz Xena. Data are available upon reasonable request.

## Abbreviations

OSCC: Oral squamous cell carcinoma
TCGA: The Cancer Genome Atlas
mIHC: Multiplex immunohistochemistry
BLCLs: B lymphoblastoid cell lines
TLS: Tertiary lymphoid structures
TMAs: Tissue microarrays
FBS: Fetal bovine serum
GFP: Green fluorescent protein
IHC: Immunohistochemistry
H&E: Hematoxylin and eosin
PBMCs: Peripheral blood mononuclear cells
ROC: Receiver operating characteristic
AUC: Area under the curve
HNSC: Head and neck cancer
