# Supplementary material for: TCL1A-expressing B cells are critical for tertiary lymphoid structure formation and the prognosis of oral squamous cell carcinoma
Source: J Transl Med. 2024 May 19;22:477. doi: 10.1186/s12967-024-05292-7 (PMC11103841; doi:10.1186/s12967-024-05292-7)
Supplement: Supplementary file 1 — Supplementary Material 1 [file 12967_2024_5292_MOESM1_ESM.docx]

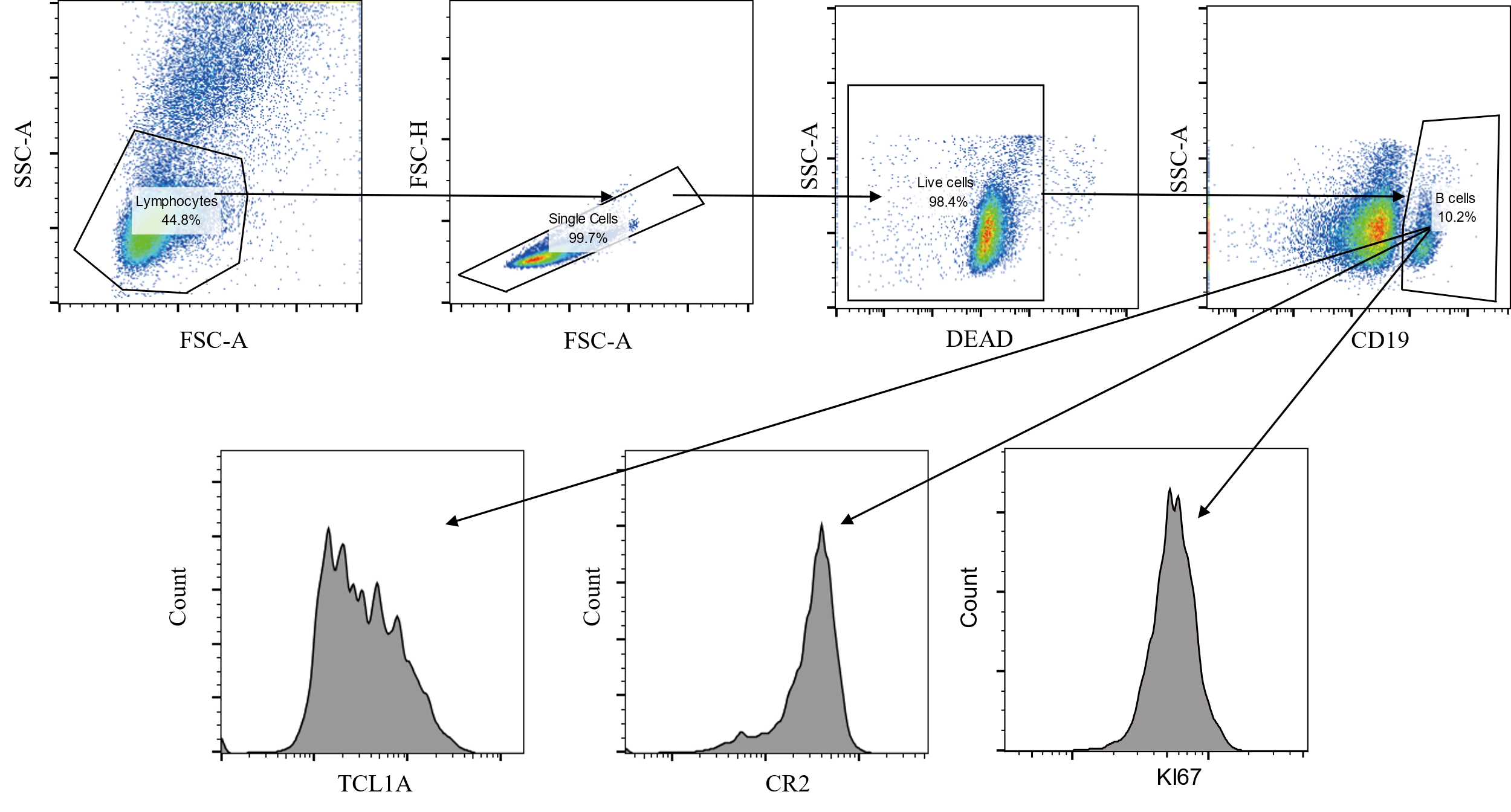


**Figure S1.** The gating strategy for flow cytometry of B cells from PBMCs.


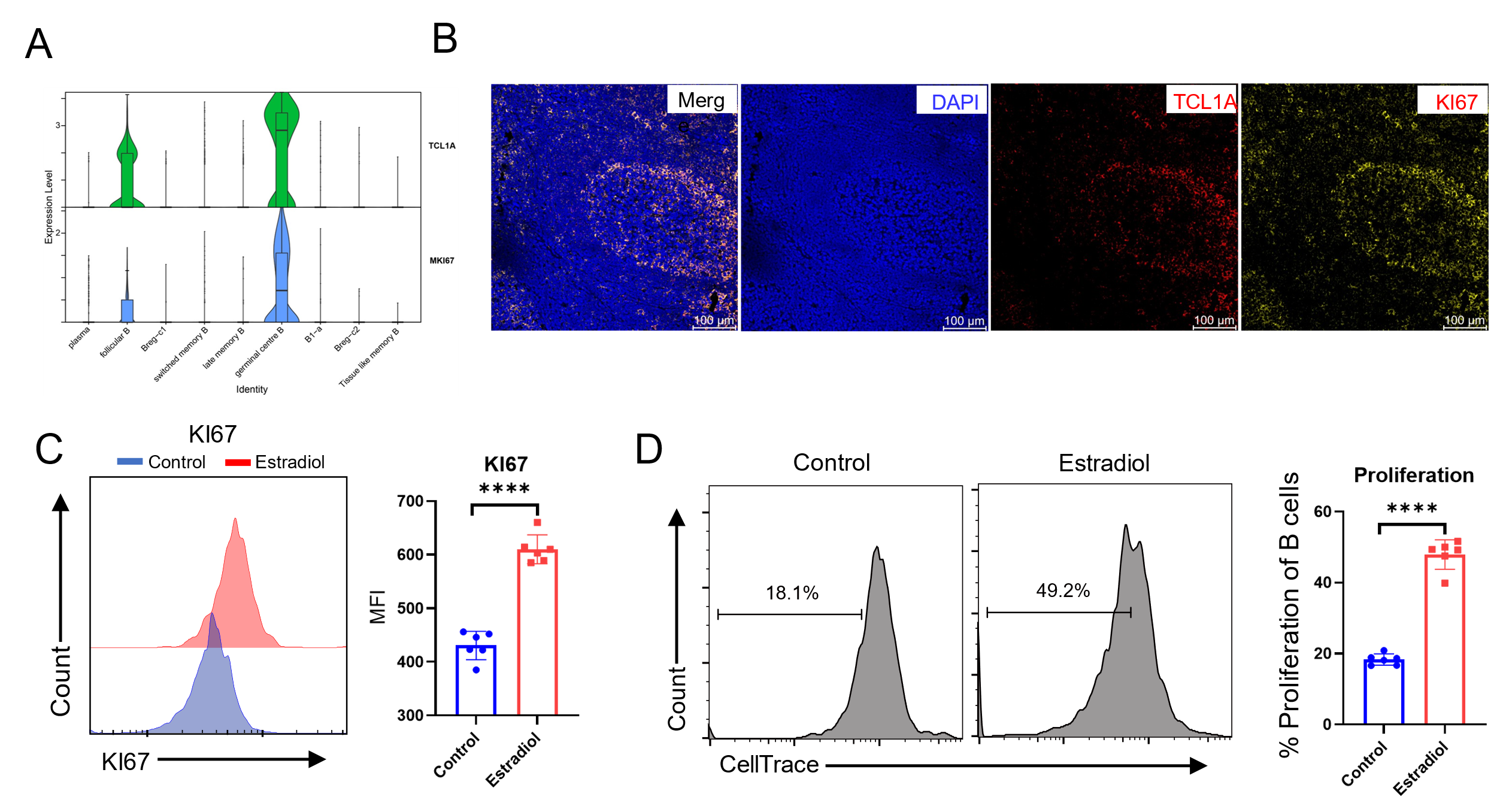


**Figure S2.** The proliferation of B cells is increased after the expression level of TCL1A is upregulated. **A** Violin plot showing that MKI67 is highly expressed in TCL1A-expressing B cells. **B** IF staining showing the expression intensity of TCL1A (red), and KI67 (yellow) in TLS of OSCC tissue (scale bars: 100 μm). **C** Flow cytometry analysis showing that the KI67 expression of B cells upregulated after B cells were treated with estradiol. **D** Flow cytometric analysis showing that the percent frequency of fast-proliferating (CellTrace^-^) cells among B cells increased after estradiol stimulation. Representative results are shown (left). The results of the quantitative analysis of the population of B cells that proliferated quickly are shown (right). Each data point represents an individual subject. **** *P* < 0.0001.


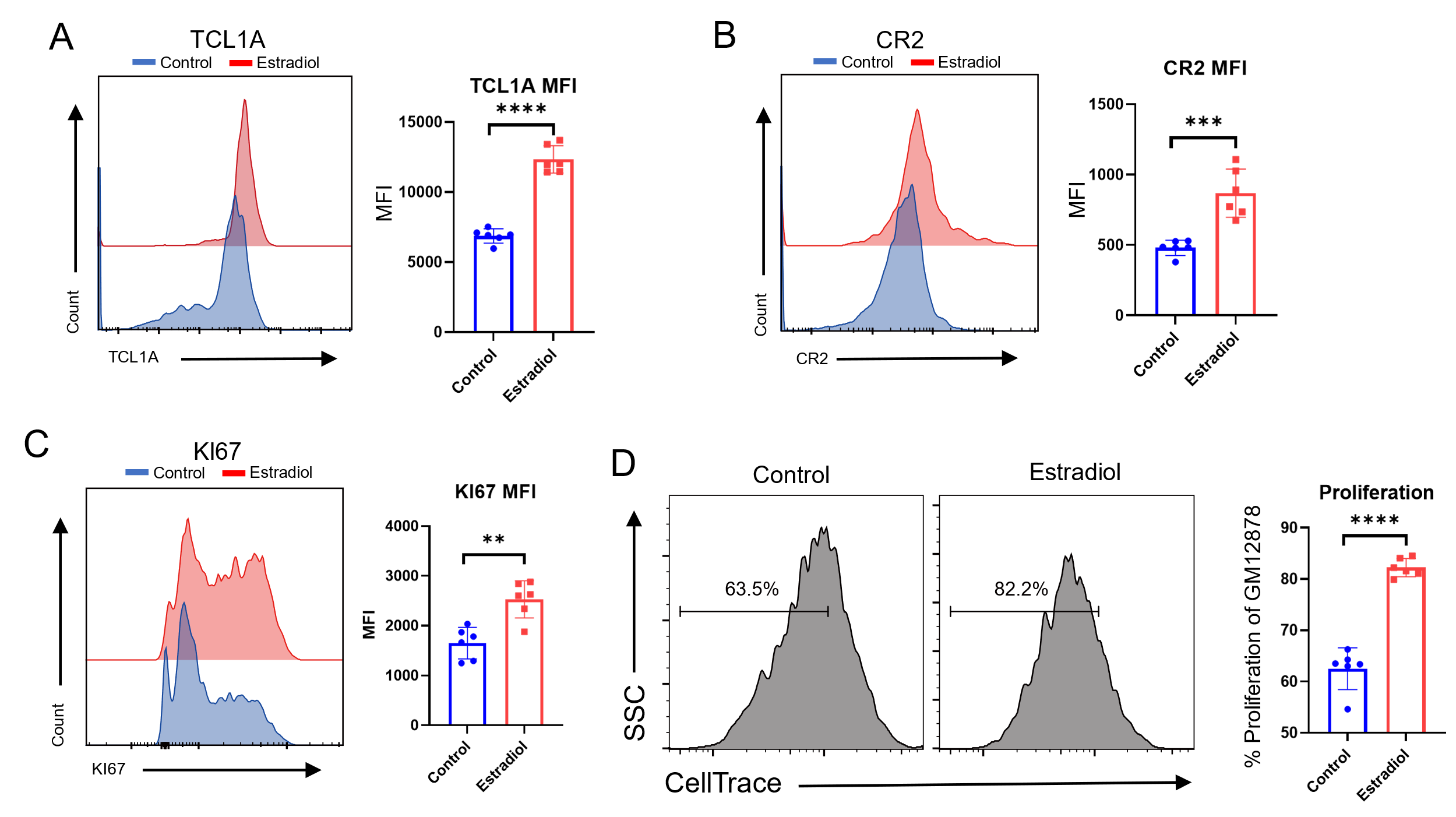


**Figure S3.** The expressions of CR2 and KI67 in BLCLs increased after the expression level of TCL1A upregulated. **A-C** Flow cytometry analysis showing that the expression of TCL1A, CR2, KI67 upregulated after BLCLs were treated with estradiol. **D** Flow cytometric analysis showing that the percent frequency of fast-proliferating (CellTrace^-^) cells among BLCLs increased after estradiol stimulation. Representative results are shown (left). The results of the quantitative analysis of the population of BLCLs that proliferated quickly are shown (right). Each data point represents an individual subject. * *P* < 0.05; ** *P* < 0.01; **** *P* < 0.0001.


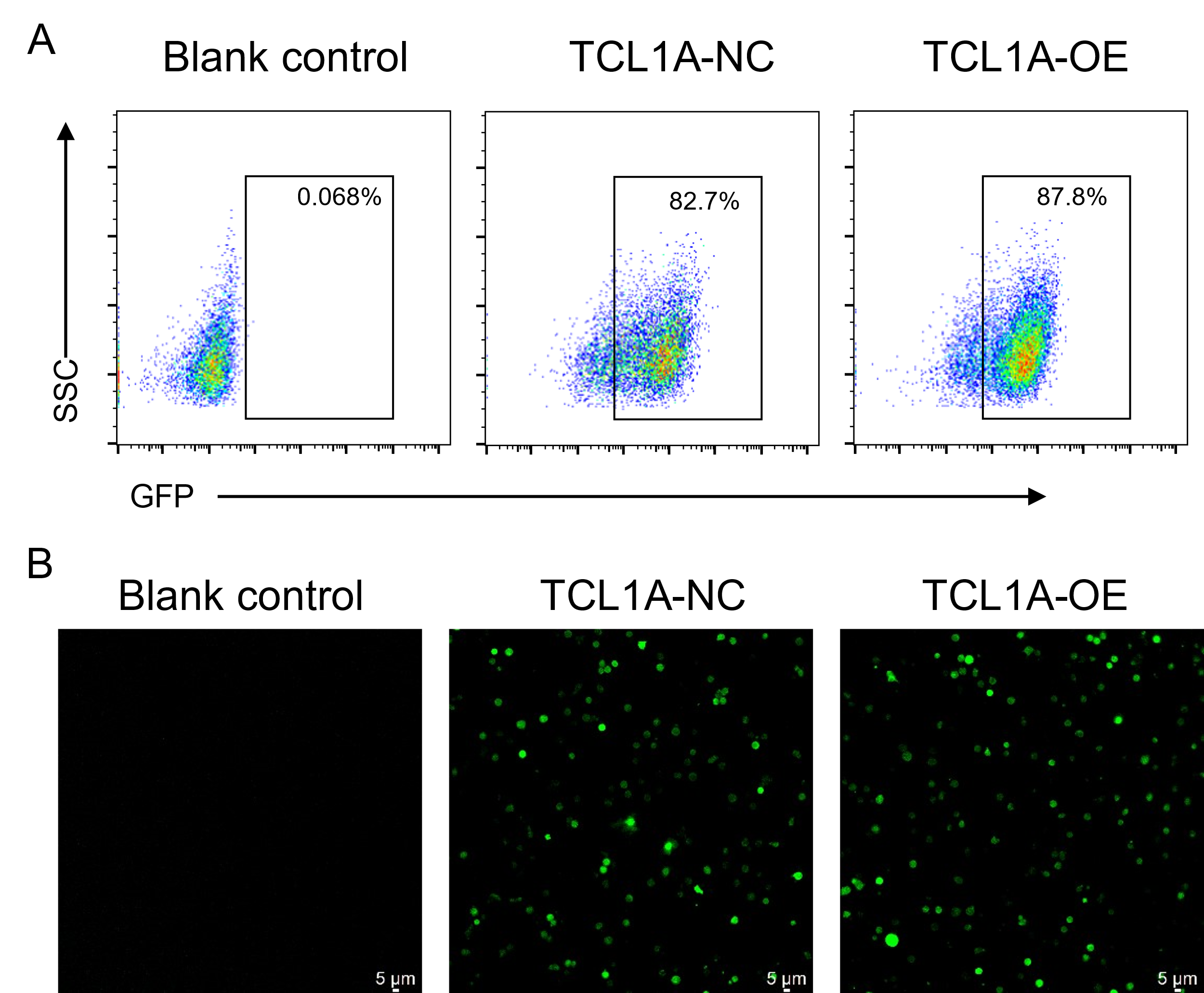


**Figure S4.** The GFP expression of the BLCLs increased after BLCLs were transduced with lentivirus. **A, B** Flow cytometry analysis and confocal microscope detection showing that the GFP expression of BLCLs increased after BLCLs were transduced with TCL1A-overexpressing lentivirus (TCL1A-OE) or control lentivirus (TCL1A-NC).

**Table S1.** Primer sequences used in this study.

| **Gene** | **Primer sequences (5'-3')** |
| --- | --- |
| TCL1A | Forward, TGCCTATCATGTGGCAGCTC |
|  | Reverse, GAGAAGCATGTCCTCCACGC |
| β-actin | Forward, CCTTCCTGGGCATGGAGTC |
|  | Reverse, TGATCTTCATTGTGCTGGGTG |
